# Over-the-Wire Retrieval of Infectious Hemodialysis Catheter-Related Right Atrial Thrombus Causing Recurrent Pleural Empyema and Sepsis: A Case-Based Review

**DOI:** 10.3390/jcm13226630

**Published:** 2024-11-05

**Authors:** Giuseppe Barilaro, Amedeo Galassi, Maria Chiara Gatto, Giulia Ciocci, Fabrizia Paola Fabrizio, Alessandra Cappelli

**Affiliations:** 1 Department of Internal Medicine, Sant’Eugenio Hospital, 00144 Rome, Italy; amedeo.galassi@aslroma2.it (A.G.); giulia.ciocci@aslroma2.it (G.C.); fabriziapaola.fabrizio@aslroma2.it (F.P.F.); alessandra.cappelli@aslroma2.it (A.C.); 2 Department of Cardiology, Sant’Eugenio Hospital, 00144 Rome, Italy; mariachiara.gatto@aslroma2.it

**Keywords:** catheter-related right atrial thrombus, percutaneous thrombectomy, methicillin-resistant *Staphylococcus aureus* (MRSA), pleural empyema

## Abstract

**Introduction:** Infectious catheter-related right atrial thrombus (CRAT) is a potentially fatal but often underestimated contingency associated with central venous catheter (CVC) in patients on hemodialysis. Management guidelines for CRAT are lacking, and its occurrence poses clinical challenges. Here, we describe the case of an infectious CRAT in a young patient on hemodialysis with peculiar clinical complications and perform a literature review. **Case presentation and literature review:** A 30-year-old man on hemodialysis after bilateral nephrectomy due to polycystic disease presented with hyperpyrexia resistant to broad-spectrum antibiotics. A pleural empyema caused by methicillin-resistant *Staphylococcus aureus* (MRSA) was diagnosed. Since fever persisted despite targeted antibiotic therapy, a transthoracic echocardiogram to exclude infective endocarditis was performed, showing a right atrial thrombus. CVC was promptly removed and the thrombus was aspirated through a percutaneous retrieval system. The thrombus cultural exam resulted positive for MRSA. After performing an extensive literature review, we could not find another case reporting the concomitance of these two rare complications. **Conclusions:** CRAT is a life-threatening complication in hemodialysis patients. While various treatment options exist, evidence-based guidelines are lacking, leading to individualized management strategies. Minimizing CVC use remains the best option for preventing such a complication.

## 1. Introduction

Arteriovenous fistula represents for many nephrologists the gold standard for patients with end-stage renal disease (ESRD) on hemodialysis, showing the best overall performances with lower rates of complications such as infections, thrombosis, and consequent need for hospitalization [1,2]. Not to mention, fistulae tend to last longer than other long-term access means. However, in recent years, the use of long-term tunneled catheters in hemodialysis patients has become increasingly common. Several reasons lie behind this growing habit, including, among others, a quicker and easier procedure compared to arteriovenous fistula creation, the unfeasibility of the latter due to anatomical or practical reasons, the indication for an urgent procedure in patients with acute kidney failure requiring urgent dialysis, the need for arteriovenous fistula maintenance, and, last but not least, centers’ or patients’ preferences [3,4]. Nevertheless, like fistulae, long-term tunneled venous catheters might present several drawbacks with not infrequent bad results. Poor outcomes range from acute occurrences such as incorrect positioning/catheter misplacement [5], insertion site hematoma, pneumothorax, hemothorax, and nerve injury [6], to more chronic complications such as thrombosis, infections, decrease in the duration of functional patency of future grafts [7], and increased mortality [2].

The presence of intravascular catheters such as peripherally inserted central catheter (PICC), central venous catheter (CVC), or port-a-cath is a long-recognized risk factor for thrombus formation especially in the right atrium, where it is usually located in pursuance of the better flow rates that can be achieved. Catheter-related right atrial thrombus (CRAT) has been extensively described in patients with malignancies, which often need CVC, PICC, or port-a-cath for periodic chemotherapy administration and intrinsically present a prothrombotic state [8]. A prothrombotic state is also associated with ESRD, and the occurrence CRAT after CVC positioning has been reported in patients on hemodialysis [9,10]. Right atrial thrombus is a potentially life-threatening event that is quite difficult to prevent, since it commonly goes unnoticed until the occurrence of one or more of its associated complications, such as pulmonary embolisms [11], systemic embolization in the presence of a patent foramen ovale, and cardiac dysfunction due to mechanical obstruction. As a matter of fact, CRAT can be perfectly asymptomatic until a severe complication occurs. In addition, once built up, a thrombus might represent the perfect milieu for bacterial growth, leading to infective hemodialysis CRAT, an underestimated complication, which is burdened, if untreated, with a very high mortality rate [12,13].

To date, evidence about the appropriate management of CRAT is quite limited due to the lack of large randomized controlled studies which are difficult to perform because of practical and ethical reasons in the absence of a gold standard. Reported management strategies mainly consist of antiplatelet therapy and/or anticoagulation [14], alone or associated with systemic thrombolysis and surgical thrombectomy, with varying results [13,15,16]. The appropriate CRAT management therefore represents a dilemma in clinical practice [17] and gathering information from case reports and cases series is of crucial importance to define the best strategy for addressing such a harmful contingency.

In the present article, we describe the case of a CVC-hemodialyzed patient with recurrent pleural empyema who was unresponsive to broad-spectrum antibiotics and received positive blood cultures for methicillin-resistant *Staphylococcus aureus* (MRSA), whose primum movens was identified in a septic atrial thrombus. We then present a literature review about the current evidence on management options for CRAT.

## 2. Literature Search

We performed a literature search for articles published from 1964 to September 2024. We used the MEDLINE database (PubMed, National Library of Medicine, Bethesda, MD, USA). We combined the following main keywords: hemodialysis, chronic kidney disease, catheter-related right atrial thrombus, anticoagulation, percutaneous thrombectomy, surgical thrombectomy, thromboaspiration, and thrombolysis. The reference lists of all articles were scanned for references not identified in the initial research.

## 3. Case Report

**Figure 1:**
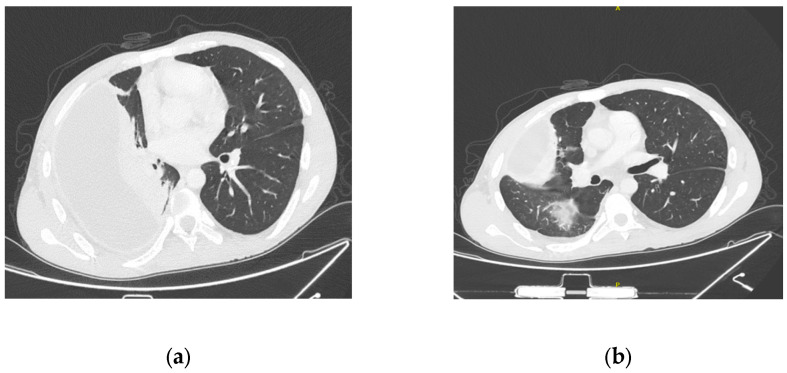
The admission chest CT scan shows a massive right pleural empyema (**a**) with pulmonary infiltrates (**b**).

A 30-year-old patient on hemodialysis after bilateral nephrectomy for polycystic disease, with a history of arterial hypertension and ostium secundum atrial septum defect previously treated with percutaneous device implantation, was sent from his hemodialysis center to the emergency room because of high-spike fever, suspecting a CVC infection as SA was isolated from swab cultures of the CVC exit site. Empiric antibiotic treatment with teicoplanin was started two days before admission. Routine chest X-rays, performed one month earlier, showed a right pleural effusion. In the emergency room, new chest X-rays were performed that, when compared with the previous exam, displayed an increase in pleural effusion with no clear pulmonary infiltrates. Blood tests showed elevated inflammatory markers with a C reactive protein (CRP) of 25.2 mg/dL. At his admission to our department, empiric gentamycin was added to teicoplanin, to cover for Gram-negative bacteria, and a thoracic computed tomography (CT) scan was performed, showing multiple pulmonary infiltrates and massive right pleural effusion suggestive of pleural empyema (Figure 1).

**Figure 2:**
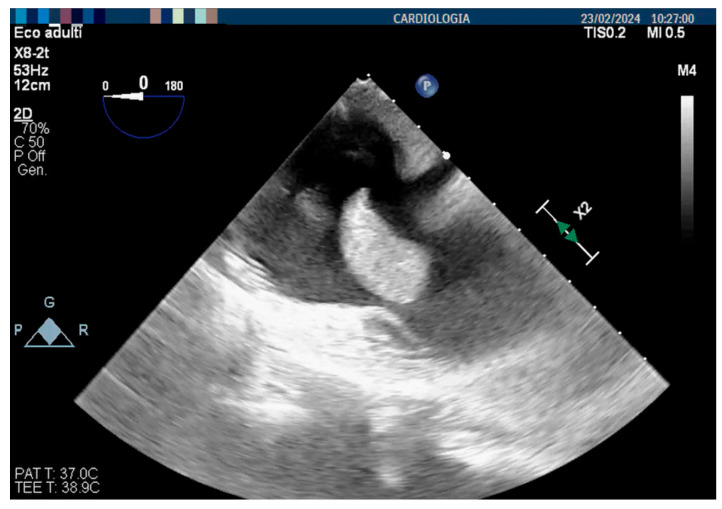
Pre-procedure transesophageal echocardiogram (TEE) showing a thrombus attached to the right atrium free wall.

Blood cultures from the CVC were obtained, which confirmed MRSA positivity. A diagnosis of community-acquired bacterial pneumonia with pleural empyema and MRSA-related CVC infection was made. A thoracic drainage was put in place, and purulent material was aspirated from the right pleura, with cultures resulting positive for MRSA. The CVC was removed and replaced, and, after the radiologic confirmation of bacterial pneumonia, antibiotic therapy was switched to vancomycin, which we decided to replace with linezolid after one week due to its better tolerability in patients with ESRD. Nevertheless, the persistence of fever and high inflammatory markers made us suspect an infective endocarditis, and a transesophageal echocardiogram revealed the presence of a thrombus attached to the free wall of the right atrium (Figure 2).

**Figure 3:**
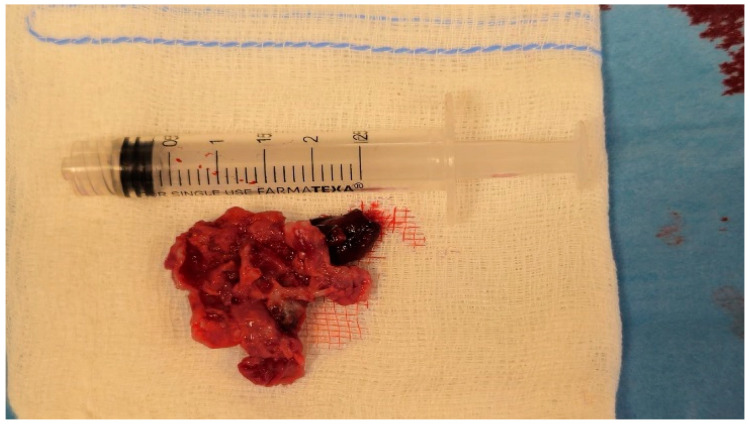
The extracted mass, with a maximum diameter of three centimeters, has a cauliflower-like shape with a prominent thrombotic component.

The multilobulated mass presented a maximum diameter of 3 cm, appeared hypermobile, and nearly occluded the tricuspid orifice. Considering the extremely elevated risk of embolization, after a multidisciplinary debate with our cardiologists and nephrologists, a decision to promptly remove the thrombotic mass was made. The clot was retrieved through the off-label use of the Inari FlowTriever System (Inari Medical, Irvine, CA, USA), an over-the-wire retrieval/aspiration system. In brief, the system consists of a support cable to which three nitinol alloy discs of different diameters are attached distally, which are opened downstream of the thrombotic formation to block and withdraw it into the guiding catheter. In order to increase the suction force, a syringe, in which a vacuum is made, is attached to the guiding catheter. The system was inserted through a 33 cm long Gore DrySeal 24 French introducer (W. L. Gore & Associates, Newark, NJ, USA), positioned through the right femoral vein. Assuming that the mass was adherent to the lateral atrial wall in continuity with the inflow tract, the guiding catheter was positioned in the superior vena cava, and the thromboaspiration maneuver was performed. After repeated attempts, the floating mass was eventually extracted. Macroscopically, the retrieved mass presented a cauliflower-like appearance, featuring a portion of fresh red thrombus and other parts covered with fibrin (Figure 3). The histological examination showed predominantly necrotic tissue with diffuse and non-specific inflammatory infiltrates, whereas the microbiological examination resulted positive for MRSA. The patient was transferred to the intensive care unit for strict monitoring, and anticoagulation therapy with unfractionated heparin was started. Congenital thrombophilia screening resulted negative, as did the search for antiphospholipid antibodies. After consulting the infectious disease specialist, considering the relatively high minimal inhibitory concentration of vancomycin, antibiotic therapy was switched to daptomycin plus fosfomycin (to cover for Gram-negative bacteria) and the patient presented progressive clinical improvement, with a reduction in high-spike fever and normalization of inflammatory markers. However, since intermittent fever persisted, we decided to perform a new CT scan, which showed pleural empyema recurrence. After consultation with thoracic surgery, we decided to perform a video-assisted thoracoscopic surgical decortication (VATSD) for the prevention of new episodes. Upon the procedure, fever completely resolved, inflammatory markers normalized, and the patient could be discharged in perfect well-being.

## 4. Discussion

Several factors predispose to CRAT occurrence in ESRD patients on hemodialysis, some of them related to the clinical condition itself, whereas others to external factors. For instance, ESRD has long been recognized as a prothrombotic state and, in the case of endothelial injury during catheter insertion, the risk of thrombus formation is nothing but negligible. ESRD is also burdened by an increased risk of infections, which is multiplied by the presence of an internal–external device such as long-term CVC. In the present article, we describe a peculiar and informative case characterized by the association of some uncommon clinical manifestations: an infective CRAT led to recalcitrant pleural empyema and sepsis, which did not respond to broad-spectrum antibiotic therapy and needed prompt thrombus aspiration and pleural decortication to prevent fatal outcomes. To our knowledge, this is the first case reporting the association of these two rare complications. Moreover, the need for an immediate thrombus aspiration with an off-label procedure makes the case even more unique.

Thrombosis is a somewhat frequent complication of both central and peripheral venous catheters [18,19], with a prevalence of CRAT that varies among different studies, ranging from 2 to 29% of patients with CVCs [10,14,20,21]. Risk factors for CVC-associated thrombosis include, among others, prothrombotic states, such as malignancies or chronic kidney diseases, and administration through catheters of substances that can damage the endothelium, as in the case of chemotherapy, or increase fluid viscosity, as in parenteral nutrition [16]. According to current guidelines, the mid- to deep right atrium is the preferred site of insertion of a CVC due to the better blood flow rates that can be achieved [22]. Nevertheless, this positioning also favors the formation of CRAT due to repeated movement of CVC tips in conjunction with cardiac contraction and the low pressure in the atrium, factors that increase the prothrombotic state. In line with this hypothesis, there are reports of increased incidence of CRAT when the catheter tip is advanced up to the right atrium, compared to when it is located in the superior vena cava [23,24].

CRAT has been sporadically described in hemodialysis patients, but it is gaining growing attention because of the worldwide increasing use of long-term CVC. In fact, although arteriovenous fistula remains the procedure of choice for patients with ESRD on hemodialysis, many centers are prone to opt for long-term CVC for several reasons, including patient preference. In a recent national survey performed in Italy, which included more than fifteen thousand subjects, the rate of CVC use in hemodialysis patients was found to be 34.5% [25], reporting an increasing prevalence in comparison with a previous study published in 2018, which showed a prevalence of around 24% [26]. This tendency poses clinical challenges, since long-term CVC has been historically associated with worse outcomes in terms of infection, cardiovascular events, and overall mortality compared to arteriovenous fistulae [27]. On the other hand, a very recent Chinese retrospective study involving elderly patients, which used propensity score matching as a group comparison technique, did not show differences in all-cause mortality between the two procedures [28]. The aforementioned Italian survey highlighted the need for formal and shared vascular access monitoring protocols to improve CVC’s long-term outcomes [29].

Right atrial thrombosis is a potentially life-threatening contingency, with a mortality rate of up to 100% if left untreated [15]. The management of such occurrence is complicated by the lack of evidence on the best treatment options, due to the absence of large, controlled studies. A systematic review and meta-analysis published in 2012, which included all cases of CRAT in hemodialysis patients reported up to December 2010 [16], identified 71 cases and categorized them into four groups based on management approaches: no treatment, systemic thrombolysis, anticoagulation, and surgical thrombectomy. Overall mortality was about 18% and predictors of mortality were advanced age, associated complications, and the decision of not removing the CVC. In one case included in the last group, the thrombus was removed through percutaneous thrombectomy.

Aspiration thrombectomy has been successfully used in both adults and preterm infants [30,31,32]. The FlowTriever^®^ system was the first to receive FDA approval for the treatment of acute pulmonary embolism [33]: by positioning a guide wire in the pulmonary artery, it is possible to advance the system to the affected area, and rapid thrombus removal is achievable through the use of three metal disks (available in different sizes). In recent years, few cases of removal of intra-atrial masses using this system have been described [34,35]. In our case, the decision to resort to this off-label procedure was due to the hypermobile appearance of the large intra-atrial mass that likely originated at the opening of the superior vena cava, posing an extremely high risk of massive pulmonary embolism, a life-threatening event. The need for a prompt intervention, after carefully considering the risk–benefit of the procedure, led us to prefer this modality over open surgery. This option should be considered in similar cases by experienced operators.

Besides thrombosis, the main complication associated with long-term CVC placement is infection, a worrisome concern in patients with end-stage renal disease on hemodialysis, who are immunocompromised. For some reason, infections seem to be less frequent in brachiocephalic vein CVCs compared to femoral CVCs [36]. Staphylococci are the most frequently reported pathogens in cases of bacteremia [16,37], and can lead to life-threatening complications such as septic embolism with pneumonia and septic shock. In our case, the infective agent was MRSA, which can be extremely hard to eradicate through antibiotic therapy in the presence of an infectious source as difficult to reach as a thrombus [38,39]. CVC infection was also complicated by a recurrent pleural empyema, which reoccurred despite several antibiotic regimens, forcing us to the ultimate decision of performing a surgical pleurodesis to prevent new episodes.

Management of infected CRATs can be very tricky, since there are no high-quality studies that performed a head-to-head comparison of different treatment modalities. In the meta-analysis mentioned above, the authors proposed an operating algorithm based on their findings, which might be of great help in daily practice [16]. The first step is CVC removal, as it triggers both thrombosis and infection. In fact, there is a bidirectional mechanism in which thrombosis serves as nidus for bacterial colonization, and infection favors a thrombogenic milieu. Suggestive evidence for infection-induced CVC thrombosis comes from the presence of neutrophil extracellular traps in the CVC fibrin sheaths [40]. Whether the egg or the hen comes first is of secondary importance, and the external trigger must be eliminated, unless it is not possible. Nonetheless, CVC removal alone has shown a slight association with mortality [16], and it must be accompanied by other measures.

Anticoagulation with unfractionated or low-molecular-weight heparin imbricated with vitamin K antagonists (VKA) should be started as soon as CRAT is diagnosed. In case of a large, mobile thrombus adherent to the catheter tip, a therapeutic anticoagulation target should be achieved before proceeding to CVC removal. Oral anticoagulation should be continued until complete dissolution of the thrombus. A special consideration must be made regarding direct oral anticoagulants (DOACs), due to their favorable risk–benefit profile and the lack of need for international normalized ratio (INR) monitoring. Several studies assessed their efficacy in intracardiac thrombosis in comparison to VKA [41,42], showing a good efficacy–safety profile which led to their endorsement as an alternative treatment by the American Heart Association [43]. Moreover, they have been used with good results in patients with congenital heart diseases in which the risk of thrombosis is increased by altered hemodynamics and the management of intracardiac thrombi can be particularly challenging [44]. However, DOACs have not been extensively studied in patients with ESRD and most international societies suggest individualized decision-making and demand further trials.

In case of contraindications to anticoagulation, the presence of very large thrombi (>6 cm of diameter), cardiac abnormalities, or valvular damage for which surgery is indicated, surgical thrombectomy—or, as an alternative in the first two cases, percutaneous removal—becomes the preferred option, with reported outcomes similar to anticoagulation [9,16,31]. Systemic thrombolysis has shown poor results in hemodialysis patients with CRAT, and presents a high risk of hemorrhage, being an option in case of failure of other strategies or in the presence of massive pulmonary embolism [16].

Although CVC replacement seems to be imperative in most cases, it is worth mentioning a small case series including seven non-infectious CRAT events in five hemodialysis patients, in which Rossi and colleagues used a conservative approach consisting of systemic anticoagulation along with injection of high-dose urokinase locking solution into the catheter dead space at the end of each hemodialysis session, without CVC removal, obtaining complete thrombus disappearance in all cases [45]. This attitude is especially convenient in cases in which alternative sites for vascular access are limited and removing the CVC is not a viable option.

Even though our case is unique, it still constitutes a single case report, which limits the generalizability of our findings about the risk factors for CRAT formation and the best treatment options. For instance, the patient herein described, a young man, does not represent the typical hemodialysis population; moreover the clot was retrieved through the off-label use of the FlowTriever System^®^, an over-the-wire retrieval/aspiration device licensed for the treatment of acute pulmonary embolism, which is not widely available. However, although it is not possible to draw definite conclusions on its efficacy for CRAT, such approach deserves thorough consideration in case of contraindications for anticoagulation, or in patients with large thrombi and high risk of pulmonary embolism that need a prompt intervention. Further studies, hopefully with a prospective design and a large number of patients, are warranted to test for its broader applicability.

On the other hand, the case that we present here is extremely peculiar: reporting the very uncommon association of two infrequent manifestations, it provides insights into currently rare but possible complications in hemodialytic patient with long-term CVC and their potential management. In this sense, CVC replacement, percutaneous aspiration of the thrombus by experienced personnel, and containment of infection sources are the three strategies to be implemented concurrently. If CVC removal is particularly difficult or there is a high risk of embolization or other perioperative complications, a conservative approach with anticoagulation and urokinase locking solution might be considered [45]. In any case, time is vital in such a life-threatening situation and prompt action should not be delayed.

## 5. Conclusions

CVC-related thrombosis is an uncommon but potentially life-threatening complication in patients with ESRD undergoing hemodialysis through central vascular access. Evidence-based guidelines for managing catheter-related right atrial thrombi are lacking, leading to treatment dilemmas in clinical practice. While CVC replacement, anticoagulation, and percutaneous or surgical thrombus aspiration, along with antibiotic treatment in cases of infection, might be effective, minimizing the use of long-term CVCs for hemodialysis remains the most effective approach to prevent CRAT. In cases of persistent fever besides targeted antibiotic therapy, a localized difficult-to-treat infection such as pleural empyema must be ruled out.

## 6. Future Directions

Since CRAT management lacks international guidelines, prospective studies comparing the different management options, with a good methodology and enough patients to make solid conclusions are warranted. For instance, prospective trials performing a head-to-head comparison of different strategies, such as anticoagulation alone vs. percutaneous thrombus aspiration vs. surgical thrombectomy, would be a turning point for the development of standardized treatment protocols, for which there is a tremendous need. Such trials should include an arm on direct oral anticoagulants, which have been shown to be effective for cardiac thrombi but have not been tested in CRAT. Gathering patients from different specialized centers across multiple countries would highly increase the feasibility of such a project.

## Data Availability

The original contributions presented in this study are included in the article. Further inquiries can be directed to the corresponding authors.
